# Clinical and MRI substrates of Symbol Digit Modalities Test impairment in multiple sclerosis patients with an adult- and late-onset

**DOI:** 10.1177/13524585261417265

**Published:** 2026-02-23

**Authors:** Antonia L Wenger, Elisabetta Pagani, Alessandro Meani, Paolo Preziosa, Antonio Gallo, Elisabeth Solana, Menno M Schoonheim, Christian Enzinger, Sergiu Groppa, Mario A Ocampo-Pineda, Alessandro Cagol, Matthias Weigel, Pasquale Calabrese, Ludwig Kappos, Cristina Granziera, Massimo Filippi, Maria A Rocca

**Affiliations:** Translational Imaging in Neurology (ThINk) Basel, Department of Biomedical Engineering, University Hospital Basel and University of Basel, Basel, Switzerland; Neuropsychology and Behavioral Neurology Unit, Interdisciplinary Platform Psychiatry and Psychology, Division of Molecular and Cognitive Neuroscience, University of Basel, Basel, Switzerland; Neurologic Clinic and Policlinic, MS Center and Research Center for Clinical Neuroimmunology and Neuroscience Basel (RC2NB), University Hospital Basel and University of Basel, Basel, Switzerland; Neuroimaging Research Unit, Division of Neuroscience, IRCCS San Raffaele Scientific Institute, Milan, Italy; Neuroimaging Research Unit, Division of Neuroscience, IRCCS San Raffaele Scientific Institute, Milan, Italy; Neuroimaging Research Unit, Division of Neuroscience, IRCCS San Raffaele Scientific Institute, Milan, Italy; Neuroimaging Research Unit, Division of Neuroscience, IRCCS San Raffaele Scientific Institute, Milan, Italy; Neurology Unit, IRCCS San Raffaele Scientific Institute, Milan, Italy; Vita-Salute San Raffaele University, Milan, Italy; Department of Advanced Medical and Surgical Sciences, University of Campania “Luigi Vanvitelli,” Naples, Italy; Neuroimmunology and MS Unit, Laboratory of Advanced Imaging in Neuroimmunological Diseases, Hospital Clinic Barcelona, Institut d’investigacions Biomèdiques August Pi i Sunyer (IDIBAPS) and Universitat de Barcelona, Barcelona, Spain; Department of Anatomy and Neurosciences, MS Center Amsterdam, Amsterdam Neuroscience, Amsterdam UMC, Vrije Universiteit Amsterdam, Amsterdam, The Netherlands; Department of Neurology, Medical University of Graz, Graz, Austria; Division of Neuroradiology, Vascular and Interventional Radiology, Department of Radiology, Medical University of Graz, Graz, Austria; Department of Neurology, Focus Program Translational Neuroscience (FTN) and Immunotherapy (FZI), Rhine-Main Neuroscience Network (rmn2), University Medical Center of the Johannes Gutenberg University, Mainz, Germany; Translational Imaging in Neurology (ThINk) Basel, Department of Biomedical Engineering, University Hospital Basel and University of Basel, Basel, Switzerland; Neurologic Clinic and Policlinic, MS Center and Research Center for Clinical Neuroimmunology and Neuroscience Basel (RC2NB), University Hospital Basel and University of Basel, Basel, Switzerland; Translational Imaging in Neurology (ThINk) Basel, Department of Biomedical Engineering, University Hospital Basel and University of Basel, Basel, Switzerland; Neurologic Clinic and Policlinic, MS Center and Research Center for Clinical Neuroimmunology and Neuroscience Basel (RC2NB), University Hospital Basel and University of Basel, Basel, Switzerland; Dipartimento di Scienze della Salute, Università degli Studi di Genova, Genova, Italy; Translational Imaging in Neurology (ThINk) Basel, Department of Biomedical Engineering, University Hospital Basel and University of Basel, Basel, Switzerland; Neurologic Clinic and Policlinic, MS Center and Research Center for Clinical Neuroimmunology and Neuroscience Basel (RC2NB), University Hospital Basel and University of Basel, Basel, Switzerland; Division of Radiological Physics, Department of Radiology, University Hospital Basel, Basel, Switzerland; Neuropsychology and Behavioral Neurology Unit, Interdisciplinary Platform Psychiatry and Psychology, Division of Molecular and Cognitive Neuroscience, University of Basel, Basel, Switzerland; Translational Imaging in Neurology (ThINk) Basel, Department of Biomedical Engineering, University Hospital Basel and University of Basel, Basel, Switzerland; Neurologic Clinic and Policlinic, MS Center and Research Center for Clinical Neuroimmunology and Neuroscience Basel (RC2NB), University Hospital Basel and University of Basel, Basel, Switzerland; Translational Imaging in Neurology (ThINk) Basel, Department of Biomedical Engineering, University Hospital Basel and University of Basel, Basel, Switzerland; Neurologic Clinic and Policlinic, MS Center and Research Center for Clinical Neuroimmunology and Neuroscience Basel (RC2NB), University Hospital Basel and University of Basel, Basel, Switzerland; Neuroimaging Research Unit, Division of Neuroscience, IRCCS San Raffaele Scientific Institute, Milan, Italy; Neurology Unit, IRCCS San Raffaele Scientific Institute, Milan, Italy; Vita-Salute San Raffaele University, Milan, Italy; Neurorehabilitation Unit, IRCCS San Raffaele Scientific Institute, Milan, Italy; Neurophysiology Service, IRCCS San Raffaele Scientific Institute, Milan, Italy; Neuroimaging Research Unit, Division of Neuroscience, IRCCS San Raffaele Scientific Institute, Milan, Italy; Neurology Unit, IRCCS San Raffaele Scientific Institute, Milan, Italy; Vita-Salute San Raffaele University, Milan, Italy

**Keywords:** Multiple sclerosis, late-onset multiple sclerosis, cognitive impairment, structural connectivity

## Abstract

**Background:**

Multiple sclerosis (MS) onset occurs at diverse ages. Age of onset impact on clinical, brain MRI, and cognitive profiles remains unclear. We investigated the substrates of Symbol Digit Modalities Test (SDMT) impairment in patients with late-onset MS (LOMS) (⩾45 years) compared to adult-onset MS (AOMS) (<45 years).

**Methods:**

294 AOMS and 80 LOMS patients with disease duration of maximum 6 years from symptom onset and 519 healthy controls were retrospectively included from a multicenter MAGNIMS data set. We assessed between-group differences and correlates of SDMT impairment measuring lesion volume (LV), atrophy, global, intra- and inter-hemispheric structural connectivity and disconnection indices.

**Results:**

38% LOMS were impaired on SDMT compared to 39% AOMS (*p* = 0.751). LOMS showed higher LV (FDR-*p* = 0.018), gray matter (GM) atrophy (FDR-*p* = 0.050), intra- and inter-hemispheric disconnection compared to AOMS (all FDR-*p* < 0.028). Furthermore, LOMS showed higher modularity (FDR-*p* = 0.018) and decreased density (FDR-*p* < 0.001). Substrates of SDMT impairment were intra-hemispheric disconnection, LV, clustering coefficient, mean strength and efficiency (AUC = 0.730) in AOMS and commissural ratio and GM atrophy (AUC = 0.760) in LOMS.

**Conclusions:**

Substrates contributing to SDMT impairment differ between these two distinct cohorts, being primarily driven by network dysfunction in AOMS and neurodegenerative processes in LOMS.

## Introduction

Patients with multiple sclerosis (pwMS) are usually diagnosed in their early twenties to forties, referred to as adult-onset MS (AOMS).^1^ However, an increasing number of pwMS is diagnosed later in life, as defined as late-onset MS (LOMS). LOMS clinical features are known,^2^ but the interaction between structural damage and cognitive impairment remains unclear. Cognitive impairment affects 40%–65% of pwMS^3^ with information processing speed being the most affected cognitive domain.^4^

Physiological aging in MS can have a significant effect on cognitive performance; hence some studies report higher cognitive impairment in LOMS due to age-related neurodegeneration,^5,6^ immunosenescence^7,8^ and chronic, low-grade inflammaging.^8–10^ Cognitive impairment can be either the result of physiological aging processes or of the disease or a cumulative effect of both.^9^

Connectomics studies white matter (WM) networks reconstructed using diffusion MRI,^11^ modeling the brain as a graph with gray matter (GM) regions as nodes and structural connections as edges. Connectome alterations may help identify (mal-)adaptation in MS.^11^

In pwMS, cognitive impairment is often associated with a disconnection syndrome,^12^ not necessarily related to the lesion volume, but more strictly dependent on the disconnection of WM tracts produced by lesions.

At the same time, the brain can implement compensatory and plastic mechanisms that, if not properly considered, contribute to the clinical-MRI dissociation.^13^ Recent works on structural connectivity proposed that the “conservation principle” of the overall connectivity holds in pwMS.^14^ Studies in pwMS support the notion that greater cognitive impairment and physical disability are associated to a decrease of inter-hemispheric connectivity, and that an increase in intra-hemispheric connectivity may act as a compensatory mechanism. This re-balancing could be thought as a flexible adaptation of the brain considering neuroplasticity.^14^

The Symbol Digit Modalities Test (SDMT) is a sensitive test primarily used to assess information processing speed among other related brain functions,^15^ a cognitive domain often compromised in early stages of MS.

Attempts to relate SDMT performance to structural brain features through MRI-based studies have highlighted the involvement of brain regions such as the thalamus,^16^ cerebellum,^17^ and fronto-parietal areas.^18^ The contribution of WM network integrity is supported by findings from a previous study,^19^ where measures of network efficiency, when considered alongside deep GM atrophy and lesion load, provided a better explanation of SDMT performance variability. In healthy controls, an intriguing temporal pattern has been described with aging, whereby alterations in fronto-striatal, precede changes in adjacent or connected GM regions in the association with SDMT.^20^

The characteristic progression of RRMS is initially inflammatory, primarily affecting the WM, and later becomes neurodegenerative, involving the GM. However, it remains unclear whether patients with LOMS fit along this pathological trajectory, and how this positioning influences their SDMT performance.

Considering that the SDMT encompasses multiple cognitive processes and engages in widespread neural networks, several pathological mechanisms could compromise its performance and functionality. In this study, we considered four different pathological effects: (1) focal pathology, as measured with T2-hyperintense WM lesion volume (WM-LV); (2) the proportion of disconnection; (3) neuro-axonal loss, as quantified with whole brain-, GM and WM atrophy; and (4) the connectivity status of the network of WM. Furthermore, we evaluated whether the principle of conservation of connectivity holds in AOMS and LOMS patients.

Based on these observations, the present study aimed to investigate clinical and MRI substrates that best predict SDMT impairment in LOMS and AOMS.

We hypothesize that, due to the advanced age of onset, the inflammatory component in LOMS patients is more limited compared to AOMS patients of similar short disease duration. We also hypothesize that compensatory and reparative mechanisms are less efficient in LOMS because of age-related reductions in neuroplasticity. Overall, our general hypothesis is that LOMS represents a later phase of the disease, in which clinical and MRI substrates of SDMT impairment might differ compared to AOMS.

## Patients and methods

### Study population

This was a multicenter, retrospective, and cross-sectional study involving seven European centers from the MAGNIMS network (www.magnims.eu).

To be included, pwMS had to be diagnosed according to the 2010^21^ or 2017 revised McDonald criteria.^22^ MS-onset was defined as the first appearance of clinical symptoms.^2,6^ Patients with age at MS-onset ⩾ 45 years were defined as LOMS, whereas those with an age at MS-onset > 18 and < 45 years were defined as AOMS. We selected pwMS satisfying the following inclusion criteria: maximum disease duration of 6 years between first symptoms and MRI; availability of clinical assessment and SDMT; availability of MRI sequences specified below. Healthy controls (HC) were additionally selected at each site with the same MRI protocol as pwMS. Participants with incomplete demographics, missing reference HC population, postprocessing failure, and violation of inclusion criteria were excluded. An overview of participants for each site is given in Supplemental Table 1.

### Clinical and neuropsychological assessment

Demographic and clinical data included sex, age at MRI, age at MS-onset, years of education, disease duration, and disease phenotype. Due to varying education scales across centers, a 13-year cut-off was used to distinguish higher from lower education. PwMS underwent a full neurological evaluation administered by trained neurologists at the corresponding site including the assessment of the Expanded Disability Status Scale (EDSS) and the definition of clinical phenotype. Five centers used the oral SDMT, in two centers the written SDMT was administered. SDMT impairment was considered if SDMT z-scores were below −1.5*SD* based on normative data.^23^ No significant differences in SDMT scores were observed between the two versions in unadjusted analysis (Wilcoxon rank-sum test, *p* = 0.700) or in linear regression models adjusted for age, sex, education, and center (*p* = 0.454).

### MRI acquisition

An overview of MRI acquisition parameters for each site is given in Supplemental Table 1. Brain MRI scans were obtained using 3.0 T scanners and the following sequences: (1) Fluid Attenuated Inversion Recovery (FLAIR) or dual echo turbo spin echo for LV assessment; (2) high resolution T1-weighted for atrophy assessment and (3) diffusion-weighted MRI for structural connectivity. Centralized analysis of MRI scans was conducted on images that passed a quality check to obtain T2-hyperintense WM LV and brain, GM and WM volumes normalized for head size (NBV, NGMV, and NWMV, respectively) (see Supplement).

### Structural connectivity

A detailed description of structural connectivity analysis is provided in the Supplement. Diffusion MR images were processed using PreQual pipeline^24^ for denoising, inter-scan intensity normalization and susceptibility-, eddy current-, and motion-induced artifact correction. T1-weighted images were processed using FreeSurfer (http://surfer.nmr.mgh.harvard.edu/) and the cortex parcellated with the Brainnetome Atlas (https://atlas.brainnetome.org/). In addition, using FIRST (https://fsl.fmrib.ox.ac.uk/fsl/fslwiki/FIRST) and SUIT (https://www.diedrichsenlab.org/imaging/suit.htm), segmentation of deep-GM structures and cerebellar regions were added to produce the node parcellation image (253 nodes).

**Figure 1.**
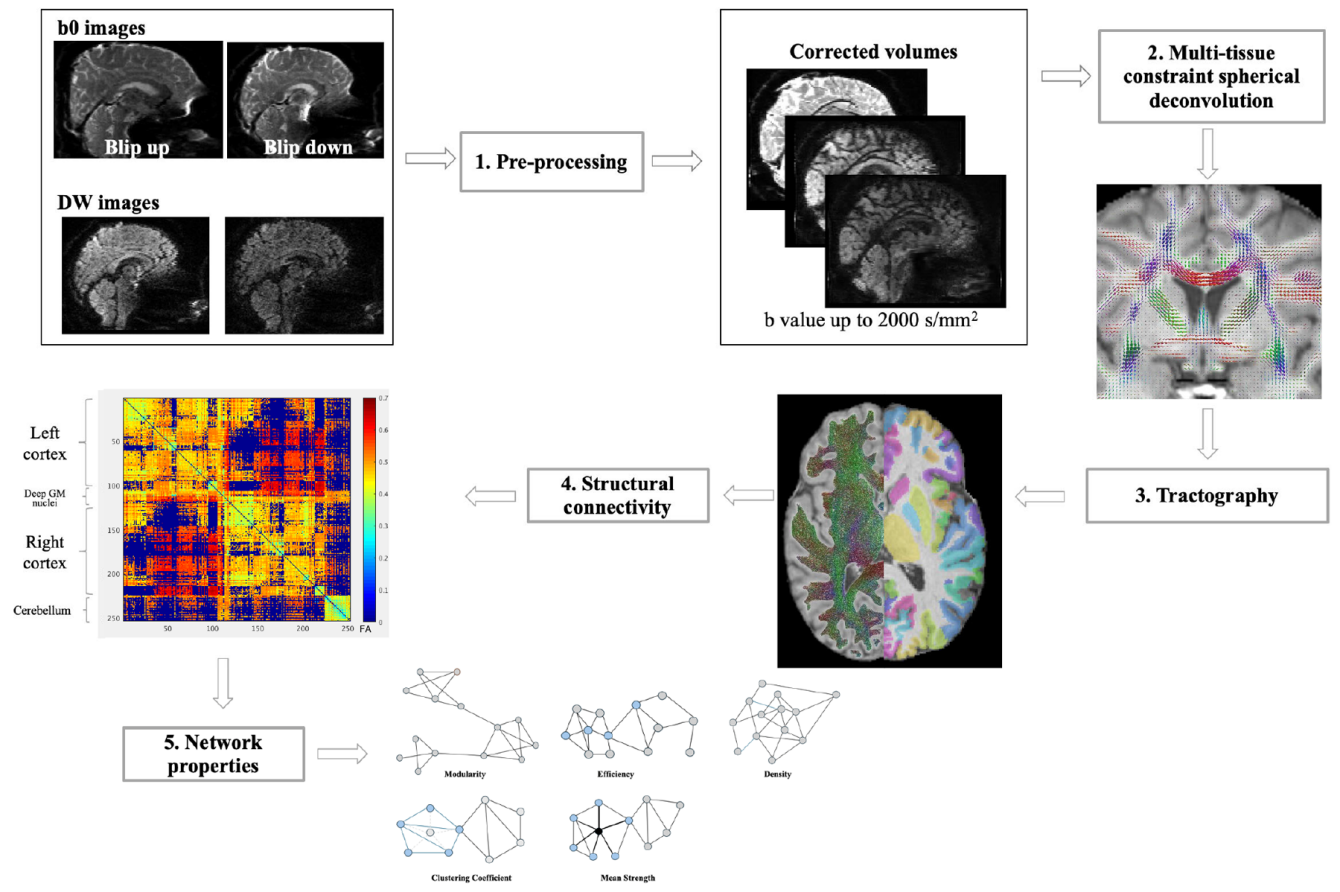
Graphical representation of the steps needed for the construction of connectivity matrices. After correction for motion and distortions (step 1), fiber orientation distribution was estimated in each voxel (step 2). Streamlines were generated with probabilistic tractography (step 3—left side) and the connectome matrices were generated (step 4) according to node parcellation images (step 3—right side). Finally, network properties were explored using the Brain Connectivity Toolbox (step 5). Refer to the methods for detailed description.

Connectomes were reconstructed using the pipeline implemented within MRtrix3.^25^ Network properties were explored using FA-weighted connectomes and brain connectivity toolbox^26^ to extract density, efficiency, mean strength, mean clustering coefficient and modularity. For a schematic representation refer to Figure 1.

**Figure 2.**
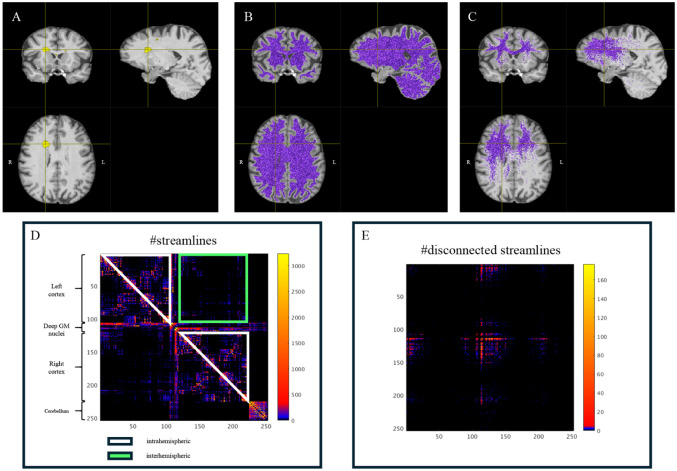
Intra- and inter-hemispheric disconnection. In the first row the figure shows in yellow an evident lesion (a), the reconstruction of all the white matter fibers (b) and the fibers that intersect the lesion (c). In the second row the connectivity matrix constructed with the number of streamlines is reported, on which the submatrix of the inter-hemispheric part is highlighted in green and that of intra-hemispheric part in white (d); the disconnectivity matrix is shown on the right (e).

A disconnection index was calculated as the percentage of streamlines intersecting lesions over total streamlines, with separate indices for supratentorial intra- and inter-hemispheric tracts (Figure 2).

Global connectivity preservation in LOMS and AOMS was investigated by evaluating intra-hemispheric efficiency and commissural ratio:^27^

1. Commissural ratio: No. of streamlines crossing hemispheres over total streamlines.
2. Intra-hemispheric efficiency: hemisphere-specific efficiency, then averaged.

The ratio of these measures was also considered.^14^

### Statistical analysis

Demographic and clinical variables were compared between groups using Chi-square, Fisher’s exact, or Mann–Whitney tests, as appropriate. We compared proportions of AOMS and LOMS patients with pathological SDMT with a Chi-square test.

LV was log-transformed and compared between AOMS and LOMS with a sex- and center-adjusted robust linear regression model. Differences in disconnection indices were assessed using quasi-binomial regression models, adjusting for sex and center. For the other MRI variables, we derived z-scores, a standardized measure of deviation from the age-, sex-, and site-specific expected reference value, based on the HC cohort, which spanned the whole age spectrum of MS patients. To this aim, we fitted linear regression models, including sex, age, the sex × age interaction term and center as covariates, to HC data. We dealt with heterogeneity in residual variances across centers by allowing heteroscedastic errors. Estimated parameters from the described models in HC were then used to convert MRI values measured in patients with MS to z-scores.

We then ran robust linear regression models to evaluate z-scores in AOMS and LOMS patients, testing the null hypothesis that mean z-scores equal zero, and to compare them between patients’ groups. False discovery correction (FDR) (Benjamini–Hochberg procedure) was applied to account for the overall number of tests.

In pwMS, the association of demographic, clinical, and MRI variables with SDMT was investigated using logistic regression models, with a robust quasi-likelihood–based estimation approach. Results were expressed as odds ratios in AOMS and LOMS patients, and differences in associations between groups were assessed through specific interaction terms. Given the imbalance in sample sizes (294 AOMS vs 80 LOMS), the analysis was repeated by applying a bootstrap resampling procedure with downsampling (*n* = 80 per group, 5000 iterations). This procedure enhanced comparability of within-group association estimates controlling for group size imbalance and assessing stability of the estimated associations and interaction effects across balanced samples. Due to their exploratory and non-conclusive nature, univariate analyses were presented for descriptive purposes, with no correction for multiple testing.

Finally, we ranked demographic, clinical and MRI variables according to their relative contribution to impairment at SDMT in AOMS and LOMS patients using a center-adjusted multivariable logistic regression with elastic net regularization. By combining ridge and lasso penalties, elastic net balances coefficient shrinkage and feature selection, enhancing model stability in the presence of correlated predictors. The regularization strength, which controls overall penalization and affects model sparsity, was tuned using the 1-standard-error rule applied to the minimum deviance obtained via 5-fold cross-validation. The analysis was repeated on 5000 bootstrap samples with downsampling (*n* = 80) to assess the stability of selected subsets. Selection percentages for each predictor were reported.

SAS release 9.4 (SAS Institute, Cary, NC, USA) and R software (version 4.3.1) were used for computations. *p*-values < 0.05 were deemed statistically significant.

## Results

### Demographic, clinical and cognitive findings

**Figure 3.**
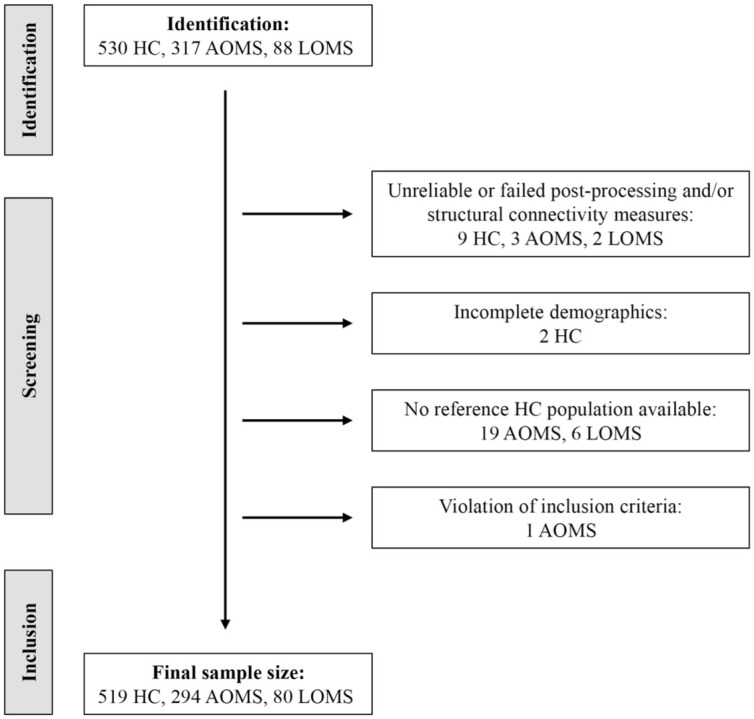
Flow chart of participants who met inclusion and exclusion criteria. Flow chart summarizing the screening, and identification process and final inclusion sample of AOMS, LOMS and healthy controls. AOMS = adult-onset multiple sclerosis; HC = healthy controls; LOMS = late-onset multiple sclerosis.

The final data set included 519 HC, 294 AOMS and 80 LOMS, as summarized in Figure 3.

Table 1 reports the main demographic, clinical and cognitive characteristics for LOMS and AOMS. Age, EDSS, and the percentage of PMS phenotype were significantly higher (all *p* ⩽ 0.039) in LOMS compared to AOMS. Mean disease duration and sex were not different between MS groups (*p* ⩾ 0.380).

**Table 1.** Main demographic characteristics of healthy controls and adult-onset and late-onset MS patients and main clinical and cognitive findings in adult-onset and late-onset MS patients.

Variables		HC (*n* = 519)	AOMS (*n* = 294)	LOMS (*n* = 80)	AOMS vs HC *p*-value	LOMS vs HC *p*-value	LOMS vs AOMS *p*-value
Male (%)		230 (44)	102 (35)	32 (40)	0.007	0.469	0.380
Female (%)		289 (56)	192 (65)	48 (60)			
Mean age at MRI (SD) [years] (range)		39.4 (13.5) (18.1 -77.4)	33.9 (7.7) (18.0 -50.4)	52.9 (4.9) (45.0 -66.0)	< 0.001	< 0.001	< 0.001
Number (%) of subjects with education ³ 13 years		371 (83) (*n* = 447)	211 (72)	40 (50)	< 0.001	< 0.001	< 0.001
Mean disease duration (SD) (years)		-	2.5 (1.8)	2.5 (1.9)	-	-	0.887
Median EDSS (IQR)		-	1.5 (1.0; 2.0)	2.0 (1.5; 3.3)	-	-	< 0.001
MS clinical phenotypes (%): RRMS/PMS		-	283 (96)/ 11 (4)	72 (90)/ 8 (10)	-	-	0.039
**Neuropsychological findings**							
Number of impaired patients (%):	SDMT	-	116/294 (39)	30/80 (38)	-	-	0.751

Comparisons performed by Chi-square (sex, education, cognitive impairment), Mann–Whitney (age, disease duration, EDSS), Fisher’s exact (clinical phenotype) tests. AOMS = adult-onset multiple sclerosis, LOMS = late-onset multiple sclerosis, SD = standard deviation, IQR = interquartile range, LV = lesion volume, NBV = normalized brain volume, NWMV = normalized white matter volume, NGMV = normalized gray matter volume, PMS = progressive multiple sclerosis, RRMS = relapsing-remitting multiple sclerosis, SDMT = Symbol Digit Modalities Test.

38% (30 out of 80) LOMS were impaired on SDMT compared to 39% (116 out of 294) AOMS (*p* = 0.751).

### MRI findings

#### Lesional and volumetric measures

Compared with HC, AOMS and LOMS had significantly lower NBV, NGMV and NWMV (all FDR-*p* ⩽ 0.043). Compared with AOMS, LOMS had significantly higher LV (FDR-*p* = 0.018) and lower NGMV (FDR-*p* = 0.0498), whereas NBV and NWMV did not differ (all FDR-*p* ⩾ 0.085) (Table 2).

**Table 2.** Volumetric and structural connectivity findings in adult-onset and late-onset MS patients.

Variables	AOMS		LOMS		LOMS vs AOMS
	Mean (SE)	*p* (FDR-*p*)	Mean (SE)	*p* (FDR-*p*)	*p* (FDR-*p*)
Lesion and volumetric measures					
Brain T2-hyperintense WM LV [mL]^a^	1.934 (0.154)	-	2.910 (0.415)	-	0.011 (0.018)
NBV	−0.523 (0.072)	<0.001 (<0.001)	−0.813 (0.139)	<0.001 (<0.001)	0.064 (0.085)
NGMV	−0.455 (0.064)	<0.001 (<0.001)	−0.746 (0.123)	<0.001 (<0.001)	0.036 (0.0498)
NWMV	−0.331 (0.073)	<0.001 (<0.001)	−0.307 (0.141)	0.030 (0.043)	0.881 (0.906)
**Network metrics**					
Density	−0.979 (0.092)	<0.001 (<0.001)	−1.667 (0.177)	<0.001 (<0.001)	<0.001 (0.001)
Efficiency	−1.071 (0.080)	<0.001 (<0.001)	−1.136 (0.153)	<0.001 (<0.001)	0.706 (0.747)
Mean strength	−1.299 (0.098)	<0.001 (<0.001)	−1.690 (0.188)	<0.001 (<0.001)	0.067 (0.085)
Clustering coefficient	−1.050 (0.084)	<0.001 (<0.001)	−1.180 (0.161)	<0.001 (<0.001)	0.474 (0.536)
Modularity	0.568 (0.076)	<0.001 (<0.001)	0.994 (0.149)	<0.001 (<0.001)	0.011 (0.018)
**Inter-hemispheric disconnection (%)**	8.38 (0.55)		11.18 (1.28)		0.028 (0.041)
**Intra-hemispheric disconnection (%)**	1.70 (0.12)		2.41 (0.29)		0.010 (0.017)
**Commissural ratio**	−0.705 (0.076)	<0.001 (<0.001)	−0.779 (0.146)	<0.001 (<0.001)	0.654 (0.712)
**Intra-hemispheric efficiency**	−0.092 (0.062)	0.138 (0.170)	0.003 (0.119)	0.977 (0.977)	0.478 (0.536)
**Commissural ratio/Intra-hemispheric efficiency**	−0.590 (0.074)	<0.001 (<0.001)	−0.763 (0.143)	<0.001 (<0.001)	0.284 (0.339)

AOMS = adult-onset multiple sclerosis, LOMS = late-onset multiple sclerosis, SE = standard error, LV = lesion volume, NBV = normalized brain volume, NWMV = normalized white matter volume, NGMV = normalized gray matter volume. Mean estimated z-scores, related standard errors, and *p*-values testing the null hypothesis that mean z-score equals zero (i.e. healthy population expected value) in AOMS and LOMS groups. *p*-values of between-group comparisons are also provided. Analyses were performed using robust linear regression models. T2-hyperintense WM LV were compared by a sex- and center-adjusted robust linear model. Disconnection indices were evaluated using sex- and center-adjusted quasi-binomial regression models and reported as estimated mean percentages. FDR correction (Benjamini–Hochberg procedure) was applied to account for the overall number of tests.

a Analysis performed on log-transformed data. Estimated means and standard errors reported are back-transformed on the original scale using delta method.

#### Whole-brain connectivity

Compared with HC, AOMS and LOMS had significantly lower density, efficiency, mean strength, clustering coefficient and increased modularity (all FDR-*p* < 0.001). Compared with AOMS, LOMS had significantly lower density (FDR-*p* < 0.001) and increased modularity (FDR-*p* = 0.018) (Table 2).

#### Inter- and intra-hemispheric connectivity

Increased disconnection was observed in LOMS compared to AOMS patients, both in inter- and intra-hemispheric connections (all FDR-*p* < 0.028). Compared to HC, AOMS and LOMS showed a significant reduction in commissural ratio and commissural ratio/intra-hemispheric efficiency index (all FDR-*p* < 0.001), with no differences between the two patient groups (all FDR-*p* > 0.339). Intra-hemispheric efficiency remained stable across groups (all FDR-*p* > 0.170). Using robust linear regression,^27,28^ commissural ratio and intra-hemispheric efficiency were negatively associated across all groups: HC (β =−0.18, SE = 0.04, *p* < 0.001), AOMS (β =−0.21, SE = 0.04, *p* < 0.001), and LOMS (β =−0.25, SE = 0.09, *p* = 0.004), with no evidence of heterogeneity (interaction *p* = 0.797).

#### Univariable associations with SDMT

Potentially distinct association profiles emerged within each group and were similarly observed in the balanced bootstrap analysis, albeit with the expected reduction in statistical power in AOMS due to downsampling (Table 3).

**Table 3.** Univariable associations of demographic, clinical, and MRI characteristics with impairment at SDMT in adult-onset and late-onset MS patients.

Variable	Full sample analysis					Balanced bootstrap analysis					
	AOMS		LOMS		LOMS vs AOMS	AOMS		LOMS		LOMS vs AOMS	
	OR (95% CI)	*p*	OR (95% CI)	*p*	*p*	percentile 95% CI	*p*	percentile 95% CI	*p*	percentile 95% CI	*p*
Age at MRI	0.99 (0.96, 1.02)	0.703	1.04 (0.95, 1.14)	0.406	0.364	(0.93, 1.06)	0.84	(0.95, 1.18)	0.376	(0.94, 1.20)	0.399
Sex (male vs female)	0.76 (0.46, 1.26)	0.288	1.00 (0.40, 2.52)	1	0.614	(0.27, 2.04)	0.572	(0.36, 2.70)	0.994	(0.32, 5.61)	0.713
Disease duration (first symptoms)	0.99 (0.86, 1.13)	0.858	1.14 (0.89, 1.45)	0.307	0.326	(0.74, 1.29)	0.926	(0.89, 1.50)	0.304	(0.80, 1.68)	0.443
Brain T2-hyperintense WM LV	1.05 (1.02, 1.07)	<0.001	1.04 (1.00, 1.09)	0.061	0.843	(1.00, 1.18)	0.069	(0.99, 1.13)	0.080	(0.88, 1.08)	0.792
NBV	0.70(0.58, 0.86)	<0.001	0.83 (0.57, 1.21)	0.337	0.434	(0.44, 1.00)	0.054	(0.53, 1.22)	0.328	(0.68, 2.19)	0.523
NGMV	0.80 (0.65, 0.98)	0.031	0.66 (0.42, 1.06)	0.087	0.481	(0.52, 1.20)	0.259	(0.39, 1.09)	0.098	(0.42, 1.59)	0.553
NWMV	0.71 (0.58, 0.87)	<0.001	1.05 (0.74, 1.51)	0.775	0.061	(0.44, 1.01)	0.061	(0.71, 1.56)	0.788	(0.87, 2.78)	0.144
Density	0.73 (0.64, 0.84)	<0.001	0.87 (0.70, 1.08)	0.222	0.181	(0.45, 0.94)	0.015	(0.67, 1.17)	0.266	(0.82, 2.06)	0.324
Mean strength	0.68 (0.59, 0.80)	<0.001	0.88 (0.70, 1.11)	0.291	0.074	(0.42, 0.88)	0.003	(0.65, 1.32)	0.397	(0.86, 2.41)	0.196
Efficiency	0.64 (0.53, 0.77)	<0.001	0.85 (0.64, 1.14)	0.289	0.098	(0.38, 0.87)	0.006	(0.57, 1.39)	0.412	(0.81, 2.65)	0.229
Clustering coefficient	0.64 (0.54, 0.77)	<0.001	0.86 (0.65, 1.15)	0.314	0.087	(0.39, 0.86)	0.006	(0.59, 1.34)	0.412	(0.82, 2.59)	0.213
Modularity	1.27 (1.10, 1.46)	<0.001	1.08 (0.91, 1.30)	0.381	0.171	(1.00, 2.12)	0.047	(0.66, 1.34)	0.469	(0.42, 1.19)	0.302
Inter-hemispheric disconnection	1.05 (1.02, 1.08)	<0.001	1.02 (0.99, 1.06)	0.248	0.200	(1.00, 1.18)	0.062	(0.94, 1.09)	0.486	(0.85, 1.04)	0.305
Intra-hemispheric disconnection	1.38 (1.18, 1.61)	<0.001	1.15 (0.98, 1.35)	0.078	0.088	(1.07, 2.94)	0.018	(0.95, 1.74)	0.136	(0.42, 1.20)	0.247
Commissural ratio	0.71 (0.59, 0.85)	<0.001	0.71 (0.50, 1.02)	0.061	0.963	(0.44, 1.03)	0.066	(0.40, 1.06)	0.085	(0.51, 1.86)	0.987
Intra-hemispheric efficiency	1.21 (0.96, 1.51)	0.105	1.06 (0.72, 1.57)	0.767	0.582	(0.81, 2.19)	0.367	(0.61, 1.71)	0.781	(0.41, 1.63)	0.664
Commissural ratio/ Intra-hemispheric efficiency	0.70 (0.57, 0.84)	<0.001	0.72 (0.50, 1.04)	0.077	0.852	(0.44, 0.99)	0.043	(0.41, 1.08)	0.105	(0.54, 1.93)	0.888

AOMS = adult-onset multiple sclerosis, LOMS = late-onset multiple sclerosis, OR = odds ratio, CI = confidence interval, LV = lesion volume, NBV = normalized brain volume, NWMV = normalized white matter volume, NGMV = normalized gray matter volume. Results were obtained from robust logistic regression models. For sex, the odds ratio (OR) compares the odds of impairment at SDMT between male and female. For continuous variables, the OR reflects the change in odds associated with a 1 year increase in age and disease duration, a 10% increase in T2-hyperintense WM LV, a 1% increase in disconnection indices, a 1-unit increase in MRI z-scores. Bootstrap-based 95% percentile confidence intervals on the OR scale were derived from the empirical distribution of regression coefficients obtained over 5000 balanced bootstrap samples (*n* = 80 per group). This downsampling approach was applied to enhance the comparability of within-group association estimates. The association analyses of T2-hyperintense WM LV and disconnection indices were adjusted for center.

#### Multivariable associations with SDMT

Elastic net regression identified higher intra-hemispheric disconnection (OR = 1.184) and brain LV (OR = 1.161), as well as lower clustering coefficient (OR = 0.927), mean strength (OR = 0.935) and efficiency (OR = 0.948) as correlates of SDMT impairment in AOMS patients (area under the curve [AUC]= 0.730). Higher LV (OR = 1.172), lower commissural ratio (OR = 0.941) and NGMV (OR = 0.960) contributed to SDMT impairment in LOMS (AUC = 0.760). Balanced bootstrap analysis confirmed the robustness of these associations with SDMT impairment, as the identified variables ranked highest in selection frequency across 5000 replicates in both AOMS and LOMS groups (Table 4).

**Table 4.** Correlates of impairment at SDMT in adult-onset and late-onset MS.

Variable	AOMS			LOMS		
	Full sample analysis AUC = 0.730		Balanced bootstrap analysis	Full sample analysis AUC = 0.760		Balanced bootstrap analysis
	Standardized beta	Standardized OR	Relative frequency of selection (%)	Standardized beta	Standardized OR	Relative frequency of selection (%)
Age at MRI			9.2			23.5
Sex (male vs female)			13.7			18.7
Disease duration (first symptoms)			8.2			21.7
Brain T2-hyperintense WM LV	0.149	1.161	49.9	0.158	1.172	48.6
NBV			16.7			8.3
NGMV			5.1	−0.041	0.960	34.9
NWMV			27.7			20.9
Density			19.6			14.1
Mean strength	-0.067	0.935	36.7			4.4
Efficiency	-0.054	0.948	33.9			14.8
Clustering coefficient	-0.076	0.927	39.2			13.4
Modularity			9.3			12.2
Inter-hemispheric disconnection			13.4			14.5
Intra-hemispheric disconnection	0.169	1.184	51.4			24.9
Commissural ratio			7.3	−0.061	0.941	36.4
Intra-hemispheric efficiency			11.4			17.5
Commissural ratio/ Intra-hemispheric efficiency			11.0			25.2

AOMS = adult-onset multiple sclerosis, LOMS = late-onset multiple sclerosis, AUC = area under the curve, OR = odds ratio, LV = lesion volume, NBV = normalized brain volume, NWMV = normalized white matter volume, NGMV = normalized gray matter volume. Results were derived from center-adjusted multivariable logistic regression models with elastic net regularization in AOMS and LOMS patients. The regularization parameter was optimized through cross-validation, and only predictors with non-zero coefficients were retained. Standardized beta coefficients and corresponding odds ratios for these variables are presented. To assess the robustness of variable selection under balanced sample conditions, the analysis was repeated across 5000 bootstrap replicates with downsampling (*n* = 80), applying the same modeling framework. The percentage of times each variable was selected across replicates is reported. See main text for further details.

## Discussion

This study provides new information regarding structural damage (LV, volumetric measures, global, intra-and inter-hemispheric structural connectivity measures and intra- and inter-hemispheric disconnection) in two distinct MS-onset cohorts. In addition, it investigates the substrates of SDMT impairment in LOMS and AOMS.

We found that despite a similar and short (clinical) disease duration, LOMS had significantly higher LV and brain GM atrophy, intra- and inter-hemispheric disconnection, increased modularity and decreased density compared to AOMS. AOMS and LOMS however differed in the factors associated with SDMT impairment.

As expected, mean age at MRI differed substantially between AOMS and LOMS per inclusion criterium to define these cohorts. The higher EDSS score in LOMS might suggest greater neurological impairment which may reflect faster disability accumulation due to neurodegenerative burden.

Given the results obtained, which more closely reflect a neurodegenerative phase in LOMS compared to AOMS, the higher proportion of PMS phenotype appears to be consistent with this characteristic. The selection of LOMS patients was based solely on the late-onset criterion, and we believe the distribution across MS phenotypes can be considered a characteristic of this group.

Delayed diagnosis in LOMS may reflect unnoticed disease progression over years, contributing to greater structural damage. LOMS was defined based on the literature showing that 80% of MS diagnoses occurs between 20 and 40 years.^29^ Lacking a consensus on cut-off age,^30^ we chose 45 years using symptom onset and not diagnosis onset, acknowledging that symptoms may precede diagnosis by years^31^ and better reflect the biological start of the disease. To minimize confounding from disease duration, we further restricted the interval between symptom onset and MRI to a maximum of 6 years. The higher neurological impairment should be considered when interpreting structural MRI changes in LOMS.

Despite similar disease duration, LOMS showed greater structural damage, indicated by LV and more pronounced GM atrophy than AOMS. This may reflect age-related factors, including increased neurodegeneration susceptibility in elderly patients. As individuals age, their central nervous system (CNS) becomes more vulnerable to damage and less capable of repair (i.e. immunosenescence, inflammaging).^6,10,32,33^ Comorbidities and vascular risk factors (e.g. hypertension, diabetes) are more frequent in the elderly, potentially increasing MS burden^34^ and accelerating neurodegeneration. These age-related factors combined likely account for more pronounced neuro-axonal loss observed in LOMS.^33^

Compared with HC, both LOMS and AOMS showed abnormal structural connectivity. A lower network density in LOMS compared to AOMS indicates that there are fewer connections relative to the maximum possible connections suggesting more extensive damage to WM integrity, which is further corroborated by higher LV, GM atrophy and disconnection, in both inter- and intra-hemispheric connections. In line with this, we found that modularity, a network segregation measure, was increased in LOMS compared to AOMS.

However, mean strength, clustering coefficient and efficiency did not show differences between LOMS and AOMS. These network metrics describe the efficiency of communication which seemed to work in LOMS as efficiently as in AOMS patients.

In addition, we found that the hypothesized compensatory mechanism between intra-hemispheric efficiency and commissural ratio was preserved in both LOMS and AOMS, at least in the early stages of the disease. These results indicate that, despite the presence of higher structural damage, the sparing of efficient connections rather than some adaptive mechanisms of structural connectivity is present in LOMS.^14^

Multivariable analysis of substrates of SDMT impairment revealed distinct patterns: in LOMS, LV, GM atrophy and inter-hemispheric connectivity were main predictors whereas in AOMS, fiber disconnection, LV and structural connectivity were predominant. These divergent patterns align with emerging evidence on the multidimensional nature of the SDMT which in more recent views has been considered to reflect broader cognitive functions, including lexical access, visual scanning, attention and working memory.^15^ This further supports the interpretation that SDMT impairment in LOMS is primarily driven by axonal degeneration independent from disconnectivity,^35^ while in AOMS inflammation and lesions more effectively interrupt network connectivity. Importantly, this distinction emphasized the SDMT as a marker of global cognitive dysfunction, as it captures performance deficits arising from different pathophysiological mechanisms depending on the age of onset, thereby reflecting the combined effects of distinct pathophysiological mechanisms.

Study’s strengths include its substantial prevalence of LOMS patients, the use of advanced MRI techniques, and the study’s stringent inclusion criteria. Moreover, the bootstrap analyses provided support for the stability and reliability of the results, thereby enhancing generalizability of our findings.

Study’s limitations are the heterogeneous MRI protocols and sample size across centers. To best harmonize MRI data, all postprocessing steps were centralized and diffusion-weighted images with multi-shell acquisitions were limited to a b-value of 2000. In addition, statistical models included adjustments for site to provide site-specific z-scores.

Moreover, SDMT was administered in either oral or written format across centers; oral normative data were applied for all participants. Although no significant differences between oral and written administrations were observed in our data set, and analyses were adjusted for center, residual measurement bias related to format-specific normative differences cannot be fully excluded.

Future studies should investigate longitudinally the substrates of SDMT impairment and collect data on comorbidities and disease-modifying treatments. Prospective work combining structural and functional MRI could further assess compensatory mechanisms and determine whether network preservation remains stable or deteriorates over time. Finally, longitudinal analyses may clarify the temporal relationship between lesion accumulation, disconnection and cognitive performance in LOMS.

## Conclusion

Cognitive deterioration in LOMS and AOMS appears to arise from distinct underlying mechanisms—primarily neurodegenerative in the former, and predominantly inflammatory and associated with network disconnection in the latter. These findings suggest that divergent pathological processes contribute to cognitive dysfunction in MS, depending on age at disease onset.

## Supplemental Material

sj-docx-1-msj-10.1177_13524585261417265 – Supplemental material for Clinical and MRI substrates of Symbol Digit Modalities Test impairment in multiple sclerosis patients with an adult- and late-onset Supplemental material, sj-docx-1-msj-10.1177_13524585261417265 for Clinical and MRI substrates of Symbol Digit Modalities Test impairment in multiple sclerosis patients with an adult- and late-onset by Antonia L Wenger, Elisabetta Pagani, Alessandro Meani, Paolo Preziosa, Antonio Gallo, Elisabeth Solana, Menno M Schoonheim, Christian Enzinger, Sergiu Groppa, Mario A Ocampo-Pineda, Alessandro Cagol, Matthias Weigel, Pasquale Calabrese, Ludwig Kappos, Cristina Granziera, Massimo Filippi and Maria A Rocca in Multiple Sclerosis Journal

sj-docx-2-msj-10.1177_13524585261417265 – Supplemental material for Clinical and MRI substrates of Symbol Digit Modalities Test impairment in multiple sclerosis patients with an adult- and late-onset Supplemental material, sj-docx-2-msj-10.1177_13524585261417265 for Clinical and MRI substrates of Symbol Digit Modalities Test impairment in multiple sclerosis patients with an adult- and late-onset by Antonia L Wenger, Elisabetta Pagani, Alessandro Meani, Paolo Preziosa, Antonio Gallo, Elisabeth Solana, Menno M Schoonheim, Christian Enzinger, Sergiu Groppa, Mario A Ocampo-Pineda, Alessandro Cagol, Matthias Weigel, Pasquale Calabrese, Ludwig Kappos, Cristina Granziera, Massimo Filippi and Maria A Rocca in Multiple Sclerosis Journal
