# Supplementary material for: Clinical and MRI substrates of Symbol Digit Modalities Test impairment in multiple sclerosis patients with an adult- and late-onset
Source: Mult Scler. 2026 Feb 23;32(3):289–301. doi: 10.1177/13524585261417265 (PMC12953661; doi:10.1177/13524585261417265)
Supplement: sj-docx-2-msj-10.1177_13524585261417265 – Supplemental material for Clinical and MRI substrates of Symbol Digit Modalities Test impairment in multiple sclerosis patients with an adult- and late-onset [file sj-docx-2-msj-10.1177_13524585261417265.docx]

**Supplementary material**

**Structural MRI and connectivity analysis**

Centralized analysis of MRI scans was conducted on images that passed a quality check. T2-hyperintense visible WM lesions were identified on FLAIR using automated approaches or on dual-echo scans using semi-automated methods, then visually checked by expert personnel. T2-LV was calculated. Whole brain, GM and WM volumes normalized for head size (NBV, NGMV, NWMV respectively) were measured on lesion-filled T1-weighted scans using SIENAX-2.^1^

Diffusion MR images were processed using PreQual pipeline^2^ for denoising, inter-scan intensity normalization and susceptibility-, eddy current-, and motion-induced artifact correction. In case the acquisition did not include scans with reversed gradient polarity, distortion correction was obtained using Synb0-DisCo^3^ to create a susceptibility-corrected synthetic b0 volume from T1-weighted images. To reduce inter-center variability, only shells with b values less than 2000 [s/mm2] were considered for the analysis. Lesion-filled T1-weighted images were processed using FreeSurfer (http://surfer.nmr.mgh.harvard.edu/) to obtain an optimal registration of the cortex onto the standard space that could be applied to transform the brainnetome atlas (https://atlas.brainnetome.org/) back to single subject space to parcellate the cortex. Additionally, using FIRST (https://fsl.fmrib.ox.ac.uk/fsl/fslwiki/FIRST) and SUIT (https://www.diedrichsenlab.org/imaging/suit.htm), segmentation of deep-GM structures and cerebellar regions were added to the brainnetome atlas to produce the node parcellation image (253 nodes) needed to compose the structural connectivity matrix.

Connectomes were reconstructed using the pipeline implemented within MRtrix3^4^ that includes the following steps: estimate of fiber orientation distributions (FOD) using multi-tissue constrained spherical deconvolution (CSD) (in case of single shell data, the Single-Shell 3-Tissue CSD method was used); generation of streamlines with probabilistic tractography initiated from seed points dynamically set using the SIFT model and applying the Anatomically-Constrained Tractography framework; filtering to match streamline density with the FOD lobe integrals;^5^ generation of two connectome matrices from tractography and node parcellation image, counting the number of streamlines and averaging fractional anisotropy (FA) along tracts. Finally, as suggested in^6^, structural connectomes were filtered by removing connections absent in >50% of healthy controls. Network properties were explored using FA-weighted connectomes and brain connectivity toolbox.^7^

Five network properties were extracted: (i) *density,* the fraction of present to possible connections; (ii) *efficiency*, the average inverse shortest path length; (iii) *mean strength*, the average sum of edges weights that are connected to a node; (iv) *mean clustering coefficient*, the tendency of two neighboring nodes to be connected to a common node; (v) and modularity, the capability of a network to be divided into modules.^7^

Furthermore, a disconnection index was calculated by considering the percentage of streamlines intersecting lesions over the number of streamlines: lesion masks were transformed into diffusion space and sampled along streamlines to identify and count those intersecting lesions using a custom MATLAB routine.

**References**

1. Battaglini M, Jenkinson M, De Stefano N. SIENA-XL for improving the assessment of gray and white matter volume changes on brain MRI. *Hum Brain Mapp* 2018;39(3):1063-77. doi: 10.1002/hbm.23828 [published Online First: 20171208]

2. Cai LY, Yang Q, Hansen CB, et al. PreQual: An automated pipeline for integrated preprocessing and quality assurance of diffusion weighted MRI images. *Magn Reson Med* 2021;86(1):456-70. doi: 10.1002/mrm.28678 [published Online First: 20210203]

3. Schilling KG, Blaber J, Hansen C, et al. Distortion correction of diffusion weighted MRI without reverse phase-encoding scans or field-maps. *PLOS ONE* 2020;15(7):e0236418. doi: 10.1371/journal.pone.0236418

4. Tournier JD, Smith R, Raffelt D, et al. MRtrix3: A fast, flexible and open software framework for medical image processing and visualisation. *Neuroimage* 2019;202:116137. doi: 10.1016/j.neuroimage.2019.116137 [published Online First: 20190829]

5. Smith RE, Tournier JD, Calamante F, et al. SIFT2: Enabling dense quantitative assessment of brain white matter connectivity using streamlines tractography. *Neuroimage* 2015;119:338-51. doi: 10.1016/j.neuroimage.2015.06.092 [published Online First: 20150708]

6. Sotiropoulos SN, Zalesky A. Building connectomes using diffusion MRI: why, how and but. *NMR Biomed* 2019;32(4):e3752. doi: 10.1002/nbm.3752 [published Online First: 20170627]

7. Rubinov M, Sporns O. Complex network measures of brain connectivity: uses and interpretations. *Neuroimage* 2010;52(3):1059-69. doi: 10.1016/j.neuroimage.2009.10.003 [published Online First: 20091009]

**Supplemental Table 1. Main subject distribution and MRI sequence parameters used at the participating sites.**

|  | | **Milan I**  **(Philips)** | **Milan II**  **(Philips)** | **Basel**  **(Siemens)**  **MP2RAGE** | **Naples I**  **(GE)** | **Naples II (GE)** | **Amsterdam (GE)** | **Barcelona**  **(Siemens)** | **Mainz**  **(Siemens)** | **Graz**  **(Siemens)** |
| --- | --- | --- | --- | --- | --- | --- | --- | --- | --- | --- |
|  |  | 24 HC, 49 AOMS, 12 LOMS | 84 HC, 30 AOMS, 6 LOMS | 85 HC, 20 AOMS, 5 LOMS | 116 HC, 45 AOMS, 6 LOMS | 21 HC, 19 AOMS, 4 LOMS | 68 HC, 25 AOMS, 9 LOMS | 42 HC, 51 AOMS, 16 LOMS | 29 HC, 15 AOMS, 14 LOMS | 50 HC, 40 AOMS, 8 LOMS |
| **Brain sequence** | **Parameters** |  | | | | | | | | |
| **3D T1-weighted** | TR/TE [ms] | 25/4.6 | 7/3.2 | 5000/2.98 | 7/2.8 | 7/3 | 7.8/3 | 1800/3.01 | 1900/2.52 | 1900/2.7 |
|  | TI [ms] | - | 1000 | 700/2500 | 650 | 650 | 450 | 900 | 900 | 900 |
|  | FA [degree] | 30 | 8 | 4/5 | 8 | 9 | 12 | 9 | 9 | 9 |
|  | FOV [mm] | 230 x 184 | 256 x 256 | 256 | 256x256 | 256x256 | 172x256 | 256x256 | 256x256 | 156x256 |
|  | Pixel size [mm] | 0.9 x 0.9 | 1 x 1 | 1 x 1 | 1x1 | 1x1 | 0.9x0.9 | 0.9x0.9 | 1x1 | 1x1 |
|  | # slices | 220 | 204 | 176 | 166 | 206 |  | 240 | 192 | 176 |
|  | Slice thickness [mm] | 0.8 | 1 | 1 | 1.2 | 1 | 1 | 0.94 | 1 | 1 |
|  | Orientation | Axial | Sagittal | Sagittal | Sagittal | Sagittal | Sagittal | Sagittal | Sagittal | Sagittal |
| **3D FLAIR/ dual echo TSE** | TR/TE [ms] | 2599/16,80 | 4800/270 | 5000/386 | 3060/24,128 | 7000/133 | 8000/125 | 5000/397 | 5000/388 | 5000/393 |
|  | TI [ms] | - | 1650 | 1800 | - | 1909 | 2350 | 1800 | 1800 | 1800 |
|  | FA [degree] | 90 | 40 | Varying refocusing flip angle | 90 | 90 |  | 90 |  | 120 |
|  | FOV [mm] | 240 x 240 | 256 x 256 | 256 x 256 | 240x240 | 256x256 |  | 256x256 | 256x256 | 256x256 |
|  | # slices | 50 | 192 | 176 | 44 | 148 |  | 192 | 192 | 176 |
|  | Slice thickness [mm] | 3 | 1 | 1 | 3 | 1.4 | 1.2 | 0.94 | 1 | 1 |
|  | Orientation | Axial | Sagittal | Sagittal | Axial | Sagittal | Sagittal | Sagittal | Sagittal | Sagittal |
| **Diffusion-weighted** | Single or multi-shell | Single shell | Multi shell | Multi shell | Single shell | Single shell | Single shell | Single shell | Single shell | Multi shell |
|  | Original b-values [s/mm^2^] | 900 | 700, 1000, 2855 | 700, 1000, 2000, 3000 | 1000 | 2000 | 1000 | 1000 | 900 | 800, 1700, 2500 |
|  | b-values used [s/mm^2^] | 900 | 700, 1000 | 700, 1000, 2000 | 1000 | 2000 | 1000 | 1000 | 900 | 800, 1700 |
|  | # DW- directions | 35 | 6, 30, 60 | 6, 20, 45, 66 | 32 | 64 | 30 | 60 | 30 | 32, 64, 96 |
|  | #DW-directions used | 35 | 6, 30 | 6, 20, 45 | 32 | 64 | 30 | 60 | 30 | 32, 64 |
|  | #b0 | 1 | 10 | 24 | 1 | 5 | 5 | 1 | 1 | 16 |
|  | Voxel size [mm] | 2.14 x 2.69 x 2.3 | 2.14 x 2.69 x 2.3 | 1.8 x 1.8 x 1.8 | 2.5 x 2.5 x 2.5 | 1.95 x 1.95 x 2 | 2 x 2 x 2.4 | 1.5 x 1.5 x 1.5 | 2 x 2 x 2.5 | 1.5 x 1.5 x 1.5 |
|  |  |  |  |  |  |  |  |  |  |  |

Abbreviations: AOMS= adult-onset Multiple Sclerosis; DW= diffusion-weighted; FA= flip angle; FOV= field of view; HC= healthy controls; LOMS = late-onset Multiple Sclerosis; ms= millisecond; NA= not applicable; TR= repetition time; TE= echo time; TI= inversion time; 3D= three-dimensional.

**Note:** For the connectivity analysis, multi-shell acquisitions with b-values higher than 2000 were excluded (please refer to the columns ‘b-values used’ and ‘#DW-directions used).
